# Memory Specificity and Mindfulness Jointly Moderate the Effect of Reflective Pondering on Depressive Symptoms in Individuals With a History of Recurrent Depression

**DOI:** 10.1037/abn0000027

**Published:** 2015-02-02

**Authors:** Kate Brennan, Thorsten Barnhofer, Catherine Crane, Danielle Duggan, J. Mark G. Williams

**Affiliations:** 1 Department of Psychiatry, Warneford Hospital, University of Oxford

**Keywords:** rumination, reflection, mindfulness, memory specificity, depression

## Abstract

In previously depressed individuals, reflective thinking may easily get derailed and lead to detrimental effects. This study investigated the conditions in which such thinking is, or is not, adaptive. Levels of mindfulness and autobiographical memory specificity were assessed as potential moderators of the relationship between reflective thinking and depressive symptoms. Two hundred seventy-four individuals with a history of three or more previous episodes of depression completed self-report measures of depressive symptoms, rumination—including subscales for reflection and brooding—and mindfulness, as well as an autobiographical memory task to assess memory specificity. In those low in both mindfulness and memory specificity, higher levels of reflection were related to more depressive symptoms, whereas in all other groups higher levels of reflection were related to fewer depressive symptoms. The results demonstrate that the relation between reflective pondering and depressive symptoms varies depending on individual state or trait factors. In previously depressed individuals, the cognitive problem-solving aspect of reflection may be easily hampered when tendencies toward unspecific processing are increased, and awareness of mental processes such as self-judgment and reactivity is decreased.

In many patients, depression takes a recurrent, and increasingly self-perpetuating course. An important factor determining the trajectory of the disorder is the way in which patients respond to negative mood. Rumination, that is, repetitive self-focused thinking about the causes and consequences of one’s negative mood, has been shown to play a central role in the processes that exacerbate and extend transient negative mood states. Ruminators are significantly more likely to develop Major Depression even when controlling for previous levels of depression (Nolen-Hoeksema, 2000; Spasojevic & Alloy, 2001), and there is a considerable body of research supporting Nolen-Hoeksema’s (1991) prediction of increased persistence of depressive symptoms among people with ruminative response styles (e.g., Nolen-Hoeksema, McBride, & Larson, 1997; Nolen-Hoeksema, Morrow, & Fredrickson, 1993; Nolen-Hoeksema & Morrow, 1991; Nolen-Hoeksema, Parker, & Larson, 1994). Maladaptive responses to negative mood seem to become increasingly important as individuals suffer from repeated episodes of depression. Risk for relapse to depression increases with number of previous episodes (Solomon et al., 2000), with relations between major life events and episode recurrence becoming increasingly more difficult to detect, because of both stress sensitization and increased autonomy of maladaptive cognitive processes (Monroe & Harkness, 2005). Cognitive research has demonstrated how, in patients with a history of recurrent depression, maladaptive ruminative patterns of thinking can be easily reinstated through even minor triggers such as subtle changes in mood (Scher, Ingram, & Segal, 2005). At the same time, maladaptive ruminative patterns of thinking seem to become increasingly habitual as individuals repeatedly elaborate on negative thoughts and feelings, reinforcing associations between negative thinking and mood (Segal, Williams, Teasdale, & Gemar, 1996). In individuals who have developed such high cognitive vulnerability for depression, attempts at reflection and cognitive problem solving may get easily derailed and lead to detrimental effects. In fact, ruminative thinking is often initiated as an attempt at cognitive problem solving (Martin & Tesser, 1996), and, despite its detrimental effects, patients frequently find it difficult to disengage from this kind of thinking, partly because of positive beliefs about its benefits (Watkins & Baracaia, 2001). It is important therefore to discern adaptive and maladaptive forms of self-focused thinking in response to negative mood, and to identify the conditions in which adaptive forms may be in danger of turning into maladaptive forms. Indeed, one of the main aims of interventions for relapse prevention in highly vulnerable patients is to help patients increase their ability to recognize and disengage from maladaptive ruminative patterns of thinking (Teasdale, 1999). In line with this clinical demand, recent research has distinguished between different facets of rumination.

In a factor analysis of the Ruminative Response Scale (RRS), the most widely used instrument for assessing ruminative tendencies, Treynor, Gonzalez, and Nolen-Hoeksema (2003) found two distinct components emerging from the items that are not confounded with depressive symptoms: *reflective pondering,* a “purposeful turning inward to engage in cognitive problem-solving to alleviate one’s depression”, and *brooding*, “a passive comparison of one’s current situation with some unachieved standard.” Secondary analyses showed differences in the association of the two constructs with depression: although both subscales showed an association with more depression concurrently, the correlation with depression was significantly smaller for reflection than for brooding. Furthermore, brooding was positively associated with depression longitudinally, but scores on the reflection measure predicted fewer symptoms at a follow-up (Treynor et al., 2003). Subsequent studies have supported these findings, and the distinction between adaptive reflective pondering and maladaptive brooding now seems well established in the literature (Burwell & Shirk, 2007; Joormann, Dkane, & Gotlib, 2006; Siegle, Moore, & Thase, 2004). However, while findings on brooding are highly consistent, accumulating data are presenting a more complex picture of the relationship between reflection and depressive symptoms (Rude, Little Maestas, & Neff, 2007).

The ‘more adaptive’ characterization ascribed to reflection was based on its more active, problem-solving approach to low mood or perceived discrepancies between current and ideal states. A recent study by Marroquin and colleagues (Marroquín, Fontes, Scilletta, & Miranda, 2010) calls into question the notion that reflection is an ‘active’ strategy per se, and found that effects of reflection were not consistently adaptive but differed depending on how active individuals’ coping styles were: in those with a less active style of coping, reflection was related to elevated levels of depression, whereas in those with a more active style of coping, this was not the case. These findings are in line with clinical perspectives suggesting that repetitive self-focused thinking, even when problem-orientated, may not always be the most adaptive approach to resolving emotional difficulties (Lyubomirsky & Nolen-Hoeksema, 1993; Nolen-Hoeksema, Wisco, & Lyubomirsky, 2008; Takano & Tanno, 2009). Furthermore, findings of two recent studies have shown reflection to predict suicidal thinking both concurrently and prospectively. Surrence, Miranda, Marroquín, and Chan (2009) found that reflection predicted suicidal ideation in individuals with a past suicide attempt, although this was not the case in participants without such a history. In a study using a large community sample, Miranda and Nolen-Hoeksema (2007) found that baseline assessment of reflection significantly predicted whether or not an individual thought about suicide at 1-year follow-up. Altogether, these data suggest that the pursuit of a more detailed understanding of the conditions in which reflection may be adaptive or maladaptive has both theoretical and clinical relevance.

Theories of modes of processing provide a useful framework for such exploration. Watkins (2008) calls attention to the fact that effects of evaluative processing can differ with regard to its level of construal, that is, whether the mental representations involved, be it of events, actions, behaviors, traits, or goals, are at a more abstract or a more concrete level. Differences in level of construal have been suggested to indicate differences in psychological distance, or distance from self (Trope & Liberman, 2010), which can be reflected on a number of different dimensions, including temporal, spatial, social, and hypothetical, as well as affective or experiential. Importantly, level of construal influences further processing. When individuals think about their negative mood at a high level of construal, this can have significant negative implications as it increases likelihood of negative judgments to be global while at the same time hampering the ability to find the solutions that are specific to a given problem (Moberly & Watkins, 2006; Watkins, 2004). Watkins has highlighted how the “why” questions that often guide brooding can lead individuals toward thinking at a higher level of construal. In fact, the tendency to generalize across situations and time is one of the defining characteristics that establish brooding as an unconstructive response to low mood (Treynor et al., 2003; Watkins, Moberly, & Moulds, 2008). It is less clear, however, where reflection fits in terms of level of construal; it is not explicit from the measure yet the distinction may have important implications for how the construct is conceptualized. It is possible that rather than being categorically abstract, the construct is sensitive to the background mode of processing and as such the relationship between reflective pondering and low mood is contingent on whether it is employed at a higher or lower level of construal. One goal of the current study was therefore to test whether level of construal moderates the relation between reflective pondering and depressive symptoms.

A domain in which level of construal is likely to be particularly consequential, both in terms of its impact on cognitive functioning and affect, is the retrieval of autobiographical memories. Research on autobiographical memory retrieval in depression has brought forth considerable evidence to suggest that the way in which patients remember autobiographical events is as important as what they remember—the actual content of the memory (Williams et al., 2007). Depressed patients show deficits in remembering autobiographical events in a specific way. When asked to remember events that refer to a particular time and place they often respond with memories that are overgeneral, by virtue of referring to a whole class of events, or with reference to semantically related content that does not include any autobiographical memory. These deficits play a pivotal role in cognitive vulnerability as they causally impact on important aspects of cognitive functioning such as social problem solving (Williams, Chan, et al., 2006), and have been shown to be a significant predictor of the overall course of the disorder (Sumner, Griffith, & Mineka, 2010). Failures to retrieve specific memories can map onto the more general dimension of psychological distance, and therefore relate to levels of construal, in a number of ways, including deficits in construing concrete representations in terms of how concretely memories relate to a particular time or place, and also in terms of affective distance. Previous research has found that overgeneral retrieval often serves to avoid the sharper affect associated with more specific memories (Raes, Hermans, de Decker, Eelen, & Williams, 2003), altogether suggesting that memory specificity might serve as a helpful indicator of level of construal and psychological distance.

However, abstractness versus concreteness may not be the only relevant dimension in the current context. Indeed being very concrete, or specific, is not necessarily protective in and of itself. Watkins (2008) points out that valence, both in terms of content, but also, and more importantly, in terms of the emotional valence that arises from appraisals of the particular content is crucial in determining the outcome of this style of thinking. Brooding involves self-judgment and self-evaluation, which in the context of negative mood is likely to have negative implications (Joormann et al., 2006; Watkins & Teasdale, 2004). Reflection mainly implies a form of processing that involves bringing inner experiences to the fore, yet the tone of this evaluation may remain neutral and objective; that is, items on the RRS do not inherently indicate any self-judgment or critical evaluation of the experience being reflected on. If reflective pondering were to take place in the context of a judgmental, self-critical mode of mind it would certainly have important consequences for the adaptiveness or benignity of this process (Rude et al., 2007). Reflection may begin simply as an attempt to gain insight or understanding. However, this well-intentioned effort could be derailed by a reactive or judgmental response to the content revealed, particularly in a context of negative mood when negative memories and judgments are more easily accessible.

In order to prevent reflection from turning into maladaptive repetitive thinking, individuals need to be able to recognize the points at which such drifts take place, and, if discrepancies cannot be resolved, need to be able to let go of unhelpful cognitive attempts to seek resolution and understanding. Such metacognitive skills are reflected in individuals’ general ability to be mindful (Teasdale, 1999). Mindfulness, defined as purposefully paying attention to present-moment experience in a nonjudgmental way (Kabat-Zinn, 2003), has previously been found to counter ruminative tendencies (Ramel, Goldin, Carmona, & McQuaid, 2004), and it is conceivable therefore that the ability to be mindful in daily life may represent another important prerequisite for adaptive engagement in reflective pondering. Such skills may be particularly relevant when individuals process information on a high level of construal, given the increased risk for reflection to get derailed under this condition.

In order to test these assumptions, we analyzed data from a large sample of previously depressed patients that were assessed at entry into a multicenter trial of treatments for relapse prevention. All of the participants had suffered from three or more previous episodes of depression and were in remission at the time of assessment, thus representing a sample in which ruminative patterns of thinking were likely to be easy to trigger and highly habitual. Based on the above reasoning, we assumed that abstract and unmindful processing would comprise a cognitive condition in which reflective pondering is likely to have maladaptive outcomes, and therefore examined the joint moderating effect of these factors on the relation between reflection and depressive symptoms. We hypothesized that reflective pondering would be a less adaptive process and be associated with relatively higher levels of depressive symptoms among those individuals with both reduced memory specificity and lower levels of mindfulness, whereas in all others reflective pondering should be adaptive and related to relatively lower levels of depressive symptoms.

## Method

### Participants

The current sample consisted of participants recruited to take part in the Staying Well after Depression Trial—a randomized-controlled trial comparing Mindfulness-Based Cognitive Therapy (MBCT) and a psychological control treatment (Cognitive Psycho-Education; CPE), to usual care in the prevention of relapse to depression (for the full trial protocol see Williams et al., 2010). Participants were recruited through local general practitioners and advertisements at two sites (Oxford and Bangor). Eligibility was assessed using the Structured Clinical Interview for *DSM–IV* Axis I, Research Version (SCID; First, Spitzer, Gibbon, & Williams, 2002), which was conducted by formally trained research assistants. Inclusion criteria for the trial were (a) age between 18 and 70 years, (b) a history of at least three previous episodes of depression, meeting *DSM–IV* TR criteria, two of which must have occurred within the last 5 years and one within the last 2 years, and (c) being in remission during the previous 8 weeks. Potential trial participants were deemed *not* to be in recovery or remission, and hence *ineligible*, if they reported that at least one week during the previous 8 they had experienced *either* a core symptom of depression (depressed mood, anhedonia) *or* suicidal feelings plus at least one other symptom of depression. Exclusion criteria were (a) history of schizophrenia, schizoaffective disorder, bipolar disorder, current abuse of alcohol or other substances, organic mental disorder, pervasive developmental disorder, or regular nonsuicidal self-injury, (b) positive continuing response to cognitive behavior therapy (CBT), because of the known effects of CBT in reducing risk of relapse, (c) current psychotherapy or counseling more than once a month, (d) regular meditation practice (meditating more than once per month), or (e) inability to complete research assessments through difficulty with English, visual impairment, or cognitive difficulties. As part of the trial, interviewer reliability for SCID diagnoses of depression was assessed by independent ratings of a sample of 91 follow-up interviews conducted by two independent psychiatrists, which yielded an agreement of κ = 0.74, 95% CI [0.60, 0.87] between the original assessor and the independent rater.

### Measures

#### Beck Depression Inventory—II (BDI-II; Beck, Steer, & Brown, 1996)

Self-reported severity of depressive symptoms was assessed using the Beck Depression Inventory-II (BDI-II, Beck et al., 1996), which consists of 21 groups of statements, referring to the presence of symptoms of depression over the past 2 weeks. Internal consistency in the current sample was α = .90.

#### Ruminative Response Scale (RRS; Treynor et al., 2003)

The RRS was used to assess different facets of self-reflective thinking. The scale consists of 22 items, which ask participants to rate on a 4-point scale, ranging from 1 = *never* to 4 = *always*, how much they engage in different cognitive responses when feeling low. The work by Treynor et al. (2003) has identified subscales for *depression-related rumination* (12 items, e.g., ‘think about feelings of achiness and fatigue,’ ‘think about how hard it is to concentrate’), *brooding* (five items, described as ‘moody pondering’ e.g., ‘think “why can’t I handle things better?”’), and *reflection* (five items referring to neutrally valenced pondering, e.g., ‘analyze recent events to try and understand why you are depressed’). Internal consistency in the current sample was α = .86 for depression-related rumination, α = .68 for brooding, and α = .73 for reflection, with corrected item-total correlations ranging from *r* = .41 to .69 for depression-related rumination, *r* = .36 to .52 for brooding, and *r* = .30 to .61 for reflection. In line with the general view of reflective pondering being an adaptive and brooding being a maladaptive strategy, we found significant correlations between brooding and other indicators of cognitive vulnerability, namely dysfunctional attitudes, as assessed using the Dysfunctional Attitudes Scale (Weissman, 1979), *r* = .40, *p* < .001, and experiential avoidance, as assessed using the Action and Experiences Questionnaire (Hayes et al., 2006), *r* = −.39, *p* < .001, whereas there were no significant correlations between reflection and either dysfunctional attitudes, *r* = .06, *p* = .26, or experiential avoidance, *r* = −.08, *p* = .16, thus supporting discriminant validity despite a significant positive correlation between the two scales, *r* = .31.

#### Five-Facet Mindfulness Questionnaire (FFMQ; Baer, Smith, Hopkins, Krietemeyer, & Toney, 2006)

The FFMQ was used to measure participants’ dispositional levels of mindfulness, or general tendency to be mindful in daily life. The questionnaire consists of 39 items that are rated on a 5-point scale ranging from 1 = *never or rarely true* to 5 = *very often or always true.* The factors that make up this scale are drawn from an exploratory factor analysis of several measures of mindfulness that suggested a five-factor solution, all of which are components of an overall mindfulness construct (Baer et al., 2006). *Observing* includes noticing or attending to internal and external experiences; *Describing* refers to labeling internal experiences with words; *Acting with awareness* includes focusing on one’s activities of the moment rather than placing attention elsewhere; *Nonjudging of inner experience* refers to taking a nonevaluative stance toward thoughts and feelings; *Nonreactivity to inner experience* is the tendency to allow thoughts and feelings to come and go without getting caught up in them. Whereas the five facets demonstrate adequate to good internal consistency, with alpha coefficients ranging from .75 to .91, some previous studies have indicated that the *Observing* subscale performs differently from the other subscales and does not load onto the higher-order ‘mindfulness’ construct (Baer, Smith, & Allen, 2004; Baer et al., 2006). For this reason the observe factor was not included in the total mindfulness score. Internal consistency of the overall scale in the current sample was α = .90.

#### Autobiographical Memory Task (AMT; Williams & Broadbent, 1986)

Participants’ ability to recall specific memories of events in their lives was measured using the Autobiographical Memory Test (Williams & Broadbent, 1986). In this task, participants are presented with 18 cue words and given 30 seconds in each case to recall a specific memory that occurred at a particular time and place. The words included in the task were positive, negative and neutral (six of each) presented in a fixed mixed order. Memory responses were recorded verbatim by the experimenter and also recorded on audiotape for later rating. Raters categorized responses as specific (events lasting less than a day), categoric (repeated events), extended (events lasting longer than a day), semantic associates, or as omissions in cases where participants failed to bring up a memory within the allotted time. In line with common and recommended practice for this task (Williams et al., 2007), we used the number of specific memories as the main outcome measure. Studies in depressed patients often additionally report numbers of categoric memories, as further analyses have suggested deficits in specificity in this group to be mainly because of increased retrieval of categoric memories (Williams & Dritschel, 1992). However, compared with those who are currently depressed, patients who are in remission tend to retrieve higher numbers of specific memories with numbers of unspecific responses relatively evenly distributed over the different categories (Barnhofer, Crane, Spinhoven, & Williams, 2007; Williams, Barnhofer, Crane, & Beck, 2005), thus rendering use of categoric memories as an outcome variable unpractical. In the current study, participants retrieved *M* = 9.14 (*SD* = 4.21) specific memories, *M* = 2.58 (*SD* = 2.32) extended memories, *M* = 1.98 (*SD* = 1.94) categoric memories, *M* = 1.60 (*SD* = 2.22) semantic associates, and *M* = 2.68 (*SD* = 2.34) omissions. The coding of the AMT was conducted by the four trial assessors. Assessors were trained by an expert coder, and trial joint-coding was conducted before proceeding with individual coding. Interreliability was examined based on seven percent (*n* = 19) of the sample selected at random. The data from these 19 complete autobiographical memory tasks (345 memories) were coded by all four assessors as well as an expert coder. Interrater reliability was established by comparing each rater’s scores with the expert coder. Cohen’s kappas for the four assessors ranged from .81 to .83.

## Procedure

Potential participants were screened over the phone by the recruitment team for the main inclusion and exclusion criteria and those likely to meet eligibility were invited to an initial assessment session during which the Structured Clinical Interview for *DSM–IV* was conducted and participants filled in self-report questionnaires. Eligible participants were then invited for a second baseline assessment session, close to the date of the initial assessment, in which they completed a number of cognitive tasks including the AMT. Mean number of days between the two assessments was *M* = 6.95 (*SD* = 3.98). All of the assessments relevant for the current analyses were completed before treatment.

## Ethics Statement

This study received ethical approval from the National Research Ethics Service, Oxfordshire (REC C Ref: 08/H0606/56) in July 2008. All subjects provided written informed consent before their participation in the study.

## Results

### Participant Characteristics

The sample was comprised of 276 adults (72.5% women) with a mean age of 43.5 years (*SD* = 11.9). One hundred sixty-three (59%) of the participants were in a relationship, and of these 92 (33.3% of the total sample) were married. In terms of highest educational qualifications, 105 (38.1%) participants had a postgraduate or professional qualification, 57 (20.7%) had a degree, and 40 (14.5%) had GCE advanced-level qualifications (school-leaving qualification after completing secondary or preuniversity education). Regarding employment status, 101 (36.6%) were currently in full-time employment and 54 (19.6%) were working on a part-time basis. The majority of participants (76%) identified themselves as White-British. In terms of clinical characteristics, participants reported a median number of six previous episodes of MDD, range = 3 to 77, and a mean age of onset of 20.8 years (*SD* = 10.7). One hundred and four participants (37.7%) reported that they had engaged in some form of suicidal behavior in the past, 182 (65.9%) reported past suicidal ideation and 92 (33%) reported not having experienced either ideation or behavior in the past. Assessment of comorbid diagnoses indicated that 104 (37.7%) reported a current or past anxiety disorder, and 44 (15.9%) reported a past or current substance-related disorder; 36 participants (13%) were diagnosed with an eating disorder but met recovery criteria.

### Relations Between Depressive Symptoms, Reflection, Brooding, Mindfulness and Memory Specificity

Zero-order correlations between the study variables are listed in Table 1. RRS reflection showed a significant positive correlation with RRS brooding, indicating moderate overlap between the two concepts, and a significant positive correlation with memory specificity, indicating that relatively higher levels of reflection were associated with relatively higher levels of memory specificity. There was no significant correlation between RRS Reflection and BDI-II scores. The correlation between RRS Reflection and FFMQ mindfulness was also not significant. RRS Brooding was not significantly correlated with BDI-II depression scores, but showed a significant negative correlation with FFMQ mindfulness indicating that higher levels of brooding were associated with relatively lower levels of mindfulness. Furthermore, the two potential moderating variables, AMT specific memories and FFMQ, both showed significant negative correlations with BDI-II scores, that is, indicating relatively higher levels of symptoms in those who show relatively lower levels of memory specificity and lower levels of mindfulness, respectively.[Table-anchor tbl1]

### Investigation of Moderator Effects in the Relation Between Reflection and Depressive Symptoms

In order to test the hypothesis that the reflection and depression relationship would be moderated by autobiographical memory specificity and trait mindfulness, we performed a multiple regression analysis with BDI-II depressive symptoms as the outcome and RRS reflection, AMT memory specificity and FFMQ mindfulness, and their interaction terms, as the predictors. In the first step of the analysis the predictor variables were entered, followed in the second step by the three two-way interactions between them. In the final step the three-way interaction was entered into the regression. Before the analysis the three predictors were mean-centered to aid interpretation. Using a *p* < .001 criterion for Mahalanobis distance no multivariate outliers among the cases were found. Results of the regression analysis are summarized in Table 2.[Table-anchor tbl2]

In step 1, FFMQ mindfulness emerged as a significant predictor, whereas effects of RRS reflection and AMT number of specific memories recalled were at trend levels. The overall model was significant, *F*(3, 266) = 11.80, *p* < .001, accounting for 11.7% of variance in the BDI-II. Inclusion of the three two-way interactions between the predictors in step 2 did not result in a significant increase in explained variance *F*(3, 263) = 1.08, *p* = .36, with none of the three two-way interactions reaching significance as a predictor. The overall model remained significant, *F*(6, 263) = 6.48, *p* < .001, accounting for 12.8% variation in BDI-II scores. Inclusion of the three-way interaction in step 3 yielded a significant increase in explained variance, *F*(1, 262) = 4.12, *p* < .05, with the overall model accounting for 14.2% of variance in BDI-II scores *F*(7, 262) = 6.18, *p* < .001. As before, none of the three two-way interactions emerged as significant; however, lower-order observations were qualified by a significant three-way interaction between RRS reflection, AMT memory specificity, and FFMQ mindfulness predicting BDI-II scores.

**Figure 1 fig1:**
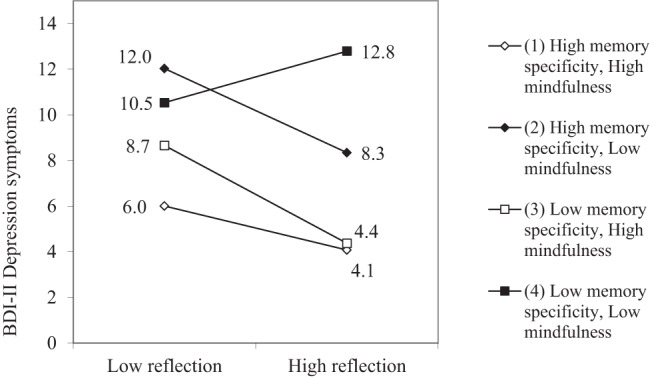
Simple slopes for the three-way interaction between reflection, memory specificity and mindfulness: predicted BDI-II total scores at low (M - 1 *SD*) and high reflection (M + 1 *SD*).

In order to probe the nature of the conditional relation, we examined the regression of BDI-II depression scores on RRS reflection at specific values of the moderators following procedures advised by Dawson and Richter (2006). The simple slopes for the prediction of BDI-II values were plotted at low and high values of the interaction variables, one standard deviation above and below the mean in each case. As can be seen in Figure 1, higher reflection was associated with *less* depression in all circumstances except when both memory specificity and mindfulness were low, where more reflection was associated with increased depressive symptoms. *T* tests of the simple slope parameters (Preacher, Curran, & Bauer, 2006) showed that all four slopes were significant at the *p* < .05 level (high memory specificity/high mindfulness: *t*(267) = −2.04, *p* = .04, *d* = .25; high memory specificity/low mindfulness: *t*(267) = −2.48, *p* = .01 *d* = .30; low memory specificity/high mindfulness: *t*(267) = −2.37, *p* = .02 *d* = .29; low memory specificity/low mindfulness: *t*(267) = 2.07, *p* = .04 *d* = .25).[Fig-anchor fig1]

Dawson and Richter’s (2006) slope difference tests were used to assess this inference empirically (see Table 3). Results showed significant differences between the slope for low memory specificity/low mindfulness and the slope for high memory specificity/low mindfulness, *t*(267) = −2.03, *p* < .05, *d* = .25, as well as the difference between the slope for low memory specificity/low mindfulness and the slope for high mindfulness/low memory specificity, *t*(267) = −2.23, *p* < .05, *d* = .27, whereas the difference between the slope for low memory specificity/low mindfulness and the slope for high memory specificity/high mindfulness was on trend levels only, *t*(267) = −1.66, *p* = .09, *d* = .20. The finding of only marginal differences between slopes for low memory specificity/low mindfulness and high memory specificity/high mindfulness is surprising considering they represent the extremes of each grouping. However, on comparing predicted BDI scores at levels of high reflection, the low memory specificity/low mindfulness and high memory specificity/high mindfulness groups show the biggest difference. Whereas high reflection relates to lowest levels of depression among those high in mindfulness and high in memory specificity, among those low in mindfulness and low in memory specificity, high reflection relates to highest levels of depression symptoms. The findings thus indicate that among those individuals low in mindfulness and low in memory specificity, levels of depression increase with levels of reflection. This pattern is not evident among the rest of the sample, which show a decrease in depressive symptoms with increased reflection.[Table-anchor tbl3]

Because the FFMQ subscales are often only moderately correlated and show differential relations with other variables, we also conducted the above regression analyses with the different subscales of the FFMQ instead of the total score entered as a predictor. For the analysis using the FFMQ subscale “Nonjudging of inner experience,” the pattern of findings replicated that from the analysis including the FFMQ total score, with RRS reflection, β = −.12, *t* = −2.09, *p* = .04, AMT specific memories, β = −.14, *t* = −2.35, *p* = .01, and FFMQ nonjudging, β = −.29, *t* = −5.09, *p* < .01, emerging as significant main effect predictors at step 1, none of the two-way interactions emerging as a significant predictor at step 2, and the three-way interaction emerging as a significant predictor at step 3, β = .20, *t* = 2.40, *p* = .02, whereas the three-way interaction did not emerge as a significant predictor in any of the analyses using the other subscales of the FFMQ. The overall model was significant at step 1, *F*(3, 267) = 11.44, *p* < .001, accounting for 11.4% of variance in BDI-II scores. Step 2 did not result in a significant increase in explained variance, *F*(3, 264) = 2.21, *p* = .09, with the whole model accounting for 13.6% of variance, whereas inclusion of the three-way interaction yielded a significant increase in explained variance at step 3, *F*(1, 263) = 5.79, *p* < .05, with the whole model accounting for 15.4% of variance.

### Investigation of Moderator Effects in the Relation Between Brooding and Depressive Symptoms

An identical regression analysis was performed, with RRS reflection replaced by RRS brooding. The model as a whole was significant, *F*(7, 264) = 4.88, *p* < .001, with AMT and FFMQ scores emerging as significant main effect predictors, *t*(264) = −2.02, *p* < .05, and *t*(264) = −4.74, *p* < .01, respectively, whereas neither RRS Brooding, *t*(264) = .05, *p* = .96, nor any of the interactions (all *p* > .10), including the three-way interaction, *t*(264) = .43, *p* = .67, provided a significant contribution. The entire model at step 1 accounted for 10.8% of variance, *F*(3, 267) = 10.74, *p* < .001. The results thus suggest that moderating effects of mindfulness and memory specificity were relevant in the relation between reflection and depression symptoms, but not in the relation between brooding and depressive symptoms.

## Discussion

Following the identification of ruminative subtypes (Treynor et al., 2003), research has mainly implicated a passive, brooding form of rumination in the exacerbation and recurrence of depressive symptoms. The other subtype, reflective pondering, conceptualized as capturing the tendency to actively seek a solution to one’s problems has been proposed as a more adaptive means of longer-term problem resolution. However, findings on the relation of reflective pondering to depressive symptoms and other emotional outcomes have been ambiguous. The current results may help to discern the conditions in which reflection may or may not be adaptive. In line with our hypotheses, reflection was associated with more depressive symptoms in those participants who showed deficits in memory specificity and described themselves as less mindful, whereas in participants who did not show deficits in both of these capacities, reflection was associated with fewer symptoms of depression.

These findings point to the susceptibility of the reflection process to become problematic under certain circumstances, and highlight the vulnerability of the reflective process to individual modes of processing. Deficits in retrieving specific autobiographical memories restrict access to details of previous experiences and may thereby increase the likelihood that earnest attempts to gain insight may fail and trigger a sense of dejection. In line with this, previous research has shown that memory overgenerality is significantly related to deficits in social problem solving (Williams, Chan, et al., 2006), and more generally that the degree to which patients are overgeneral in their autobiographical memory is associated with a more chronic course of depression (Sumner et al., 2010).

However, our findings suggest that a tendency toward abstract construal alone, as reflected in deficits in memory specificity, is not associated with a change in the direction of the relationship between reflection and depression, at least in patients who are currently relatively well. Instead, it is only when deficits in memory specificity come together with low levels of mindfulness that turning inward to reflect on one’s inner experience may result in a worsening of mood and other depressive symptoms. Our data further suggest that it is particularly the tendency to be judgmental in response to one’s experience that is problematic in this context. Mindfulness is trained as a means of increasing patients’ capacity to observe experience from a nonjudgmental stance so that they will be less likely to engage in ruminative processes about their experience and better able to recognize and disengage from maladaptive patterns of thinking. It would seem plausible that the degree to which patients can observe their experience without judgment is of particular relevance with regard to whether or not abstract reflective pondering derails toward brooding. Indeed, metacognitive awareness—the ability to observe one’s own mental activities from an observer perspective—has been argued to be the core characteristic of a mindful mode of processing (Teasdale, 1999). Whereas high levels of mindfulness, including a nonjudgmental stance, should allow individuals to see more clearly when thinking patterns become repetitive and unhelpful, a lack of mindfulness, including a more judgmental stance, puts individuals at risk of reacting impulsively and negatively to emotionally difficult content. If occurring together with a tendency to construe autobiographical events on an abstract level, such deficits in awareness of one’s own psychological responses may increase the likelihood of reflective pondering to become contaminated with brooding thoughts. In addition to its moderating role in the relation between reflection and depressive symptoms, mindfulness also showed a substantial direct association with depressive symptoms indicating a general limiting relation between the two constructs.

Interpretation of our findings needs to take into account that the incremental effect of the interaction that qualified the relation between reflection and depressive symptoms was relatively small, thus leaving room for a number of other determining factors that were not included in the current study. Furthermore, there are a number of limitations. First, participants were recruited to the study based on their history of depression and because of the nature of the clinical trial within which this study was conducted had to meet criteria for a minimum of three previous episodes of depression while at the same time being in remission at the time of entry into the study. Participants were therefore likely to have high levels of vulnerability, but showed low levels of current symptoms. Compared with community samples used in previous research (Treynor et al., 2003), distribution of depression scores was therefore more restricted in range, and the combination of vulnerability and low symptoms might have created a constellation, which was particularly conducive for observing moderator effects, but might have hindered detection of relations between the two components of the rumination construct and depressive symptoms over the entire group. Within this constellation, low levels of symptoms may have facilitated adaptive effects of reflection, whereas detrimental effects of reflection were more likely to arise only in those in whom additional factors, such as low mindfulness and low memory specificity, increased the likelihood that reflection might lead into downward cycles of negative mood and thinking. Similarly, for many participants periods of negative mood might not have been severe or long enough for brooding to exert its effect to a degree that would be reflected in a strong increase in depressive symptoms, particularly in a situation in which individuals would consider their mood as considerably better than during previous episodes of depression. There is a general tendency for the influence of determining factors to become more clearly visible at higher levels of outcome variables whereas variations at lower levels are likely to be determined by a broader range of hidden factors (Cade, Terrell, & Schroeder, 1999), a tendency that might have been particularly relevant in the current sample because of high levels of vulnerability. Further research may want to investigate in more detail the symptom threshold at which the influence of brooding becomes significant in highly vulnerable and community samples using techniques such as quantile regression (Radford et al., 2014). Second, the current study is cross-sectional. Previous research had found that beneficial effects of reflection became visible only in longitudinal observations and further research will have to use prospective designs in order to see whether mindfulness and memory specificity moderate the effects of reflective pondering on later occurrences of depressive symptoms and relapse to depression. More experimental designs might consider the use of mild standardized stressors in order to ensure presence of sufficiently potent events around which self-reflective processes might evolve (see the design of the study by Moberly & Watkins, 2006). Third, with the exception of those relating to memory specificity, the current findings are all based on self-report and therefore vulnerable to reporting biases. Future research might want to include assessments of actual thinking processes at times when individuals were feeling low in order to corroborate self-reports, for example using records from expressive writing (Watkins et al., 2004) or through use of stream-of-consciousness techniques (Pennebaker et al., 1990). Use of experimental manipulations of thinking style would allow investigating causal effects. Previous research has aimed to induce mindfulness by asking participants to process experiences in an experiential mode of thinking that emphasizes sensory-perceptual aspects of experience as compared with a narrative mode that focuses on relating to experience through conceptual thinking, an approach that follows seminal work by Nolen-Hoeksema (e.g., Lyubomirsky & Nolen-Hoeksema, 1993) and Watkins (e.g., Watkins & Teasdale, 2001), who have used experimental manipulations to induce rumination and distraction or particular aspects of a ruminative and other styles of thinking. In our own research we have induced memory overgenerality in experimental designs by manipulating the abstractness of cue words presented to participants for retrieving memories (Williams, Chan, et al., 2006), whereas other research has successfully manipulated mode of processing by using manipulations that required participants to either retrieve specific or general memories (Dalgleish et al., 2007). Future studies might want to combine manipulations on the dimensions of experiential versus narrative and abstract versus concrete processing in order to test the current findings using experimental manipulations. Previous research by Moberly and Watkins (2006), who investigated interactions between trait rumination and mode of processing by using processing mode inductions before a failure experience, might serve as a model for such research.

In terms of their implications, it is interesting to view the current findings in the context of recent trends in treatment development (Hayes, Villatte, Levin, & Hildebrandt, 2011). These have particularly highlighted the benefit of a self-focus that is less linguistic and more experiential. However, reflective thinking is a central tenet of human experience and the present study suggests that in the main, this conceptual or linguistic processing of experience is not problematic. It is important, though, to take into account the cognitive context in which reflection is employed. The current findings suggest that, in previously depressed individuals who are more concrete and specific in the creation of solutions, while mindful of their reactions to the thoughts and emotions being reflected on, reflection will not be associated with increases in symptoms. This is despite the fact that, in the context of high cognitive vulnerability, this might be a ‘risky’ means of resolving low mood. The Response Style conceptualization of reflection that was applied in this study is that of a ‘cognitive problem-solving’ process, not necessarily critical or judgmental and seemingly more active than other forms of self-focused thinking (Nolen-Hoeksema et al., 2008; Treynor et al., 2003). However, reflective thinking often does not just involve an open curiosity; it seeks understanding and meaning with the intention of resolving some difficulty or unwanted situation. Applied as a linguistic, conceptually based means of ‘solving’ sadness, the question is, how likely is it that such an approach will result in the generation of an actual solution? If this analytical-type search is exacerbated by an abstract processing style, then the likelihood of solutions being reached may reduce. Add to this a difficulty in seeing a reactive, judgmental response to what is unearthed during reflection and this response has the potential to be not merely unhelpful, but harmful.

## Figures and Tables

**Table 1 tbl1:** Summary of Intercorrelations, Means and Standard Deviations for Scores on the BDI-II, RRS Reflection, RRS Brooding, FFMQ, and AMT Number of Specific Memories

*Measure*	1	2	3	4	5
1. BDI-II					
2. RRS Reflection	−.07				
3. RRS Brooding	.08	.31**			
4. FFMQ	−.33**	−.02	−.20**		
5. AMT Specific	−.16*	.18**	.02	.08	
*M*	8.2	12.1	13.2	118.9	9.1
*SD*	8.0	3.1	3.0	18.1	4.1
*Note*. BDI-II = Beck Depression Inventory II; RRS = Ruminative Response Style Questionnaire; FFMQ = Five Facet Mindfulness Questionnaire; AMT Specific = Autobiographical Memory Task - number of specific memories.					
* *p* < .05. ** *p* < .01.					

**Table 2 tbl2:** Hierarchical Multiple Regression Analysis Predicting BDI-II Depression Scores From RRS Reflection, FFMQ Mindfulness, AMT Memory Specificity, and the Interactions of These Factors

Predictor	Δ*R*^*2*^	β	*t*	*p*
Step 1	.12			
RRS Reflection		−.12	−1.97	.05
AMT Specific Memories		−.09	−1.56	.12
FFMQ		−.33	−5.29	.00
Step 2	.01			
RRS Reflection × AMT Specific Memories		−.05	−.89	.37
RRS Reflection × FFMQ		−.07	−1.20	.23
AMT Specific Memories × FFMQ		.00	.07	.94
Step 3	.01			
RRS Reflection × AMT Specific Memories × FFMQ		.13	2.03	.04
Total *R*^*2*^	.14			
*Note*. RRS = Ruminative Response Style Questionnaire; AMT = Autobiographical Memory Task; FFMQ = Five Factor Mindfulness Questionnaire.				

**Table 3 tbl3:** T-Tests for Differences of Slopes Plotted in Figure 2

Pairs of slopes	*t*	*p*
1 vs. 2	.60	.55
1 vs. 3	.78	.44
1 vs. 4	−1.67	.09
2 vs. 3	.20	.84
2 vs. 4	−2.03*	.04
3 vs. 4	−2.23*	.03
*Note.* 1 = high mindfulness/high memory specificity, 2 = low mindfulness/high memory specificity, 3 = high mindfulness/low memory specificity, 4 = low mindfulness/low memory specificity.		
* *p* < .05.
